# Why Is Iron Deficiency/Anemia Linked to Alzheimer’s Disease and Its Comorbidities, and How Is It Prevented?

**DOI:** 10.3390/biomedicines11092421

**Published:** 2023-08-30

**Authors:** Karin Fehsel

**Affiliations:** Neurobiochemical Research Unit, Department of Psychiatry, Medical Faculty, Heinrich-Heine-University, 240629 Düsseldorf, Germany; fehsel@uni-duesseldorf.de; Tel.: +49-(0)211-922-2712; Fax: +49-(0)211-922-2733

**Keywords:** glucose transporter, insulin resistance, HIF1α, hydrogen sulfide, metabolic syndrome, ferritin, *gingko biloba*, COVID-19

## Abstract

Impaired iron metabolism has been increasingly observed in many diseases, but a deeper, mechanistic understanding of the cellular impact of altered iron metabolism is still lacking. In addition, deficits in neuronal energy metabolism due to reduced glucose import were described for Alzheimer’s disease (AD) and its comorbidities like obesity, depression, cardiovascular disease, and type 2 diabetes mellitus. The aim of this review is to present the molecular link between both observations. Insufficient cellular glucose uptake triggers increased ferritin expression, leading to depletion of the cellular free iron pool and stabilization of the hypoxia-induced factor (HIF) 1α. This transcription factor induces the expression of the glucose transporters (Glut) 1 and 3 and shifts the cellular metabolism towards glycolysis. If this first line of defense is not adequate for sufficient glucose supply, further reduction of the intracellular iron pool affects the enzymes of the mitochondrial electron transport chain and activates the AMP-activated kinase (AMPK). This enzyme triggers the translocation of Glut4 to the plasma membrane as well as the autophagic recycling of cell components in order to mobilize energy resources. Moreover, AMPK activates the autophagic process of ferritinophagy, which provides free iron urgently needed as a cofactor for the synthesis of heme- and iron–sulfur proteins. Excessive activation of this pathway ends in ferroptosis, a special iron-dependent form of cell death, while hampered AMPK activation steadily reduces the iron pools, leading to hypoferremia with iron sequestration in the spleen and liver. Long-lasting iron depletion affects erythropoiesis and results in anemia of chronic disease, a common condition in patients with AD and its comorbidities. Instead of iron supplementation, drugs, diet, or phytochemicals that improve energy supply and cellular glucose uptake should be administered to counteract hypoferremia and anemia of chronic disease.

## 1. Introduction

Dementia refers to a syndrome of progressive nature characterized by the alteration of higher cognitive functions severe enough to impair independent daily living. The prevalence of dementia is around 697 cases per 10,000 people aged ≥50 years [1], and, because lifespan prolongs, the number of individuals with dementia is likely to increase in the following decades. Moreover, the COVID-19 pandemic might also contribute to a higher prevalence of dementia in the near future [2].

Two subtypes of dementia can roughly be distinguished: vascular dementia due to previous stroke or hemorrhage and Alzheimer’s disease (AD), which is the most common chronic degenerative disease with brain tissue loss, chronic inflammatory processes, mitochondrial dysfunction, and redox imbalance. The accumulation of amyloid ß plaques and intracellular neurofibrillary tau protein tangles is one of the hallmarks of AD.

Recent studies indicate that these pathophysiological mechanisms are shared by other diseases. Beside hormonal deficiency in advanced age and the *Apolipoprotein Eε4* genotype, a wide range of diseases—such as depression, hypertension, cardiovascular disease, diabetes mellitus type 2, osteoporosis, or obesity—predispose to AD [3,4]. Identifying the relationship between AD and other diseases might help to address the complexity of AD and allow treatment of common dysregulated pathways. The significant prevalences of anemia and non-alcoholic fatty liver disease in all these disorders might present a link between them that refers to a common pathomechanism, but this link is still poorly understood.

Iron, glucose, and energy metabolism are closely intertwined [5,6]. Cells require both glucose and iron for essential functions like energy production, but their amounts within the body and in individual cells are tightly regulated. Glucose uptake is the prerequisite for sufficient ATP production along the mitochondrial respiratory chain, which consists of multiple iron-containing complexes. Iron deprivation results in a significant reduction in the abundance of these mitochondrial iron–sulfur proteins [7]. Instead, proteins involved in glucose utilization and glycolysis are upregulated under iron deficiency. Curiously, the same genes are also upregulated, at least temporarily, under impaired glucose uptake [4,8].

In the following, this review elaborates on the molecular insights that help to further understand comorbidities and find pathogenic mechanisms common to all mentioned disorders and possible links. Recent clinical and research data point to deficits in glucose and energy metabolism [9] accompanied by anemia/iron deficiency [4,10] in all these diseases.

## 2. Iron Metabolism

**Figure 1:**
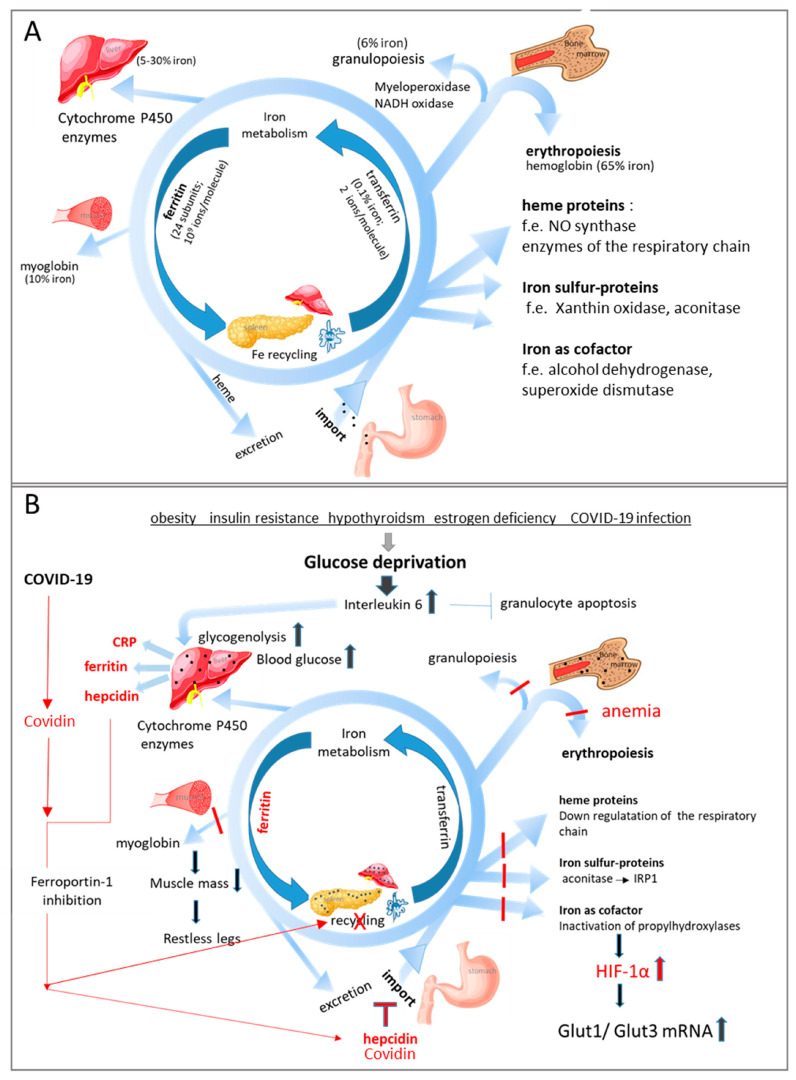
Summary of iron regulation. (**A**) Iron is either recycled from dying erythrocytes or resorbed in the duodenum. It is transported in the blood bound to transferrin, which binds the transferrin receptors expressed on all dividing body cells. After endocytosis of the whole iron/transferrin/transferrin receptor complex, the iron is liberated and intracellularly available for the synthesis of heme proteins or iron–sulfur clusters. Iron released from cells is bound to ferritin. Cells regulate their iron content by up- or downregulating transferrin receptors on their surface. Most of the iron is required in the bone marrow for erythropoiesis and in the muscle for myoglobin synthesis. (**B**) Hormone/growth factor/neurotransmitter deficiency or resistance, as well as obesity and viral infection, reduce cellular glucose uptake, resulting in a stress response. The acute phase response proteins CRP and ferritin are secreted from the liver, and the proinflammatory cytokine interleukin 6 induces glycogenolysis and hepcidin expression in the liver. This peptide reduces iron efflux from cells and duodenal absorption by promoting the degradation of the only iron exporter, ferroportin 1. Excessive iron (:::) is stored in ferritin and hemosiderin primarily in the liver and kidney. These iron deposits can be detected in liver and kidney biopsies from patients with nonalcoholic fatty liver disease and chronic kidney disease. Missing replenishment of the bone marrow with transferrin-bound iron impairs not only erythropoiesis but also granulopoiesis [12]. However, the longer survival of granulocytes induced by IL6 [13] compensates for the reduced production. Intracellular upregulation of ferritin and removal of iron stabilize the transcription factor HIF1α, which counteracts glucose deprivation by increasing the expression of Glut 1 and 3. HIF1α—hypoxia-induced factor; CRP—C-reactive protein; IRP—iron-responsive protein; Glut—glucose transporter. Arrows indicate activation of enzymes or pathways; red lines indicate inhibition of proteins or pathways.

Most of the iron is bound as pentacoordinated iron in heme proteins, such as hemoglobin, myoglobin, and cytochrome P450 enzymes. Iron homeostasis is achieved by recycling heme proteins in macrophages of the spleen, liver, kidney, and bone marrow and by import from the duodenum (Figure 1). Bound to transferrin, it is transported in the blood, and about 65% of the iron is inserted into the heme group of hemoglobin in the bone marrow. In fact, available body iron is prioritized for hemoglobin synthesis. Therefore, it is important to mention that iron deficiency in tissue cells proceeds the decline of hemoglobin and the number of erythrocytes under anemia [11].

**Figure 2:**
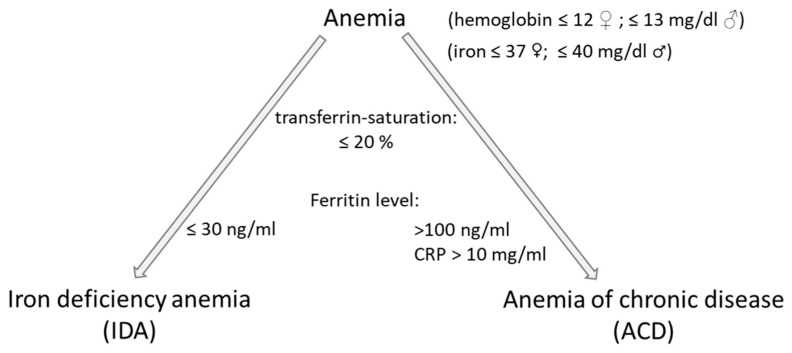
Simplified diagram for the diagnosis of anemia types based on the publication of Resal et al. [16].

Reduced erythropoietin production, long-lasting inflammation [14], obesity, hormone deficiency or resistance, or disturbed iron supply for erythropoiesis are the main triggers of anemia [10] (Figure 1B). Anemia is diagnosed when hemoglobin levels fall below 12 g/dL in women and <13 g/dL in men [15]. Two types of anemia can be distinguished: anemia of chronic disease (ACD) (also called anemia of inflammation) and iron deficiency anemia (IDA) (Figure 2). The circulatory level of serum ferritin—an iron-binding protein—acts as a gold-standard prognostic marker for iron deficiency (hypoferritinemia) and iron overload (hyperferritinemia). IDA is defined by serum ferritin levels less than 30 ng/mL and a low bone marrow iron level, while ACD is linked to serum ferritin levels higher than 100 ng/mL and elevated C-reactive protein (CRP) levels.

Ferritin is a heteropolymer with 24 subunits, which consist of heavy and light chains. The ratio of both chains is tissue- and cell-type-dependent and is altered during disease pathologies. The ferroxidase activity of the heavy chain transforms the aggressive Fe^2+^ into unreactive Fe^3+^. Thousands of Fe^3+^ iron atoms can be stored inside its hollow cavity as a hydrated ferric oxyhydroxide mineral. Hyperferritinemia is often linked with iron deposits in the liver and bone marrow, thereby increasing the risk of non-alcoholic fatty liver disease [17] and disturbed hematopoiesis [18].

In the elderly population, changes in ferritin concentration do not always reflect variations in iron stores because ferritin is an acute-phase protein and is affected by inflammatory processes irrespective of the iron store status. Transferrin saturation is less affected by inflammation and may therefore be more reliable than serum ferritin, particularly under permanent pro-inflammatory conditions called inflammaging [19].

## 3. Comorbidities of AD

Epidemiological and biological evidence supports a link between Alzheimer’s disease and type 2 diabetes mellitus [20,21,22], cardiovascular disease [22,23,24], osteoporosis [4,25], and depression [26,27].

Multimorbidity with insulin resistance, older age, and obesity are the main risk factors not only for AD but also for severe COVID-19 infection [28], with type 2 diabetes and cardiovascular disease as the leading two comorbidities that worsen COVID-19 pathology. Cognitive impairment, depression, and delirium are possible neurological manifestations of viral infection [29,30], and the long-term consequences of this infection on the prevalence of dementia cannot be estimated yet.

While the association between AD and its comorbidities, which were seen as independent diseases with different local restrictions until recently, becomes more and more important, the association of all diseases with anemia or, more often, iron deficiency, is largely unknown.

Kim et al. [31] observed an association between anemia, AD, and osteoporosis. Zarate-Ortiz et al. [32] associated lower concentrations of hemoglobin and higher body weight with an increased risk for depressive symptoms. In addition, obesity is linked to anemia in young adolescents [33] as well as impaired brain energy [34]. A special focus should be set on the association of intra-natal iron deficiency with autism, schizophrenia, and abnormal brain structure [35].

In general, dysregulated iron metabolism is associated with cognitive disorders, including memory and attention disorders, and anemia or, at least, an iron deficiency [36,37]. Ferritin levels in the cerebrospinal fluid are associated with inflammation markers, phospho-tau, and apolipoprotein E levels [38]. Amyloid precursor protein and tau have been reported to have physiological roles in neuronal iron homeostasis [39,40].

In neuropathological and neuroimaging surveys, brain iron levels have been reported to be associated with plaque and tangle pathology. Further studies reveal that iron could not only enhance the production of amyloid ß but also directly bind to amyloid ß and tau to promote their aggregations [40].

Anemia is a relatively common finding in heart failure. In a large meta-analysis of 34 studies, comprising 153,180 patients with chronic heart disease, 37.2% of them were anemic and had an increased risk of mortality [41]. The specific cause of anemia is still unknown, but for the confounders ‘iron deficiency’ and ‘inflammation,’ the strongest evidence-based data exist [42]. Indeed, in patients with heart failure, higher levels of proinflammatory cytokines and CRP are inversely related to hemoglobin levels [43]. Current treatment options include intravenous or oral iron supplementation or blood transfusions in cases of extreme anemia, but clinical trials have failed to show convincing evidence for benefits. Therefore, no feasible therapeutic strategy for the long-term management of chronic anemia in patients with chronic heart failure exists based on the risk-benefit profile [44]. Detailed insights into chronic heart disease and anemia are given in the review of Siddiqui et al. [45].

Iron regulates immune responses and influences the course of viral infections. The dysregulation of iron homeostasis associated with anemia appears to be a hallmark of severe COVID-19 infection [46]. Recent studies revealed a virus-encoded hepcidin-like peptide termed COVIDin [47] that blocks iron import and recycling (Figure 1B). Infected patients have low levels of iron, total iron binding capacity, and transferrin but significantly higher levels of ferritin and transferrin saturation [48]. The iron level is even a predictor of mortality.

Anemia due to chronic kidney disease has been studied the most. It relies on decreased production of erythropoietin by the kidneys, leading to suppression of erythropoiesis. This form of anemia cannot, therefore, be equated to iron deficiency anemia. However, it is also linked to dementia, among several other diseases [49], probably because obesity and diabetes are independent risk factors for kidney disease.

In general, iron deficiency is less noticed by clinicians, although the WHO has identified it as a public health problem since 2004. It might even point to pathophysiologic mechanisms common to a wide range of diseases. However, it is still unclear whether anemia itself is an additional driver or just a passenger in all diseases.

## 4. Glucose Deprivation Is a Common Pathogenic Mechanism

**Figure 3:**
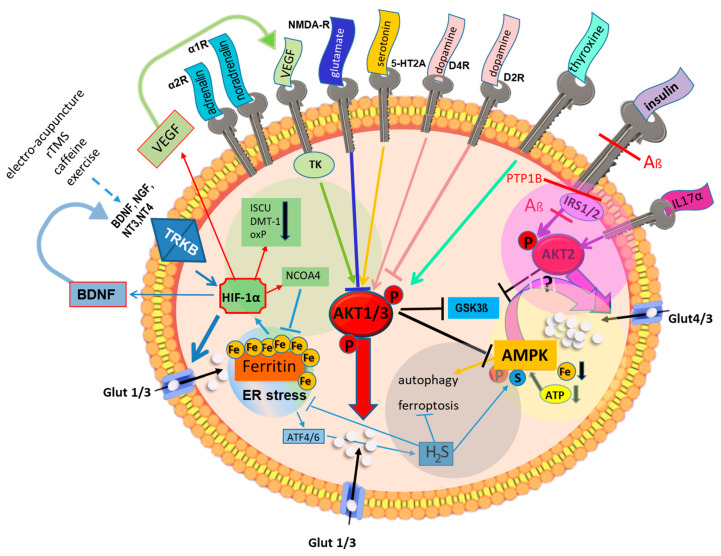
Schematic representation summarizing the regulation of cellular glucose uptake based on publication [4]. Depending on the corresponding receptors on the cell surface, cytokines, growth factors, neurotransmitters (Akt1/3), and insulin (Akt2) (magenta circle) activate the serine/threonine kinase Akt, which coordinates the translocation of Glut-loaded vesicles to the plasma membrane. Missing ligand binding to the respective receptors downregulates the Akt pathway and triggers intracellular glucose depletion. Several survival pathways are immediately initiated. Inhibition of Akt induces ER stress and the upregulation of ferritin, thereby reducing intracellular iron levels and stabilizing HIF1α (green circle). Metabolism shifts from oxidative phosphorylation (oxP) to glycolysis because the synthesis of iron–sulfur proteins in the mitochondria is downregulated. Concurrently, the expression of Glut1 and Glut3 is increased. Further loss of energy and iron activates AMPK, which fuels the cell with glucose through Glut4 translocation (yellow circle). This pathway is promoted by metformin. Another survival pathway is induced by ATF4/6 in the ER stress response. H2S increases autophagy and inhibits ER stress and ferroptosis (grey circle). Aß, beta-amyloid; AMPK, adenosine monophosphate-activated protein kinase; ATF, activating transcription factor; ATP, adenosine triphosphate; BDNF, brain-derived neurotrophic factor; DMT-1, divalent metal transporter-1; ER, endoplasmic reticulum; Fe, iron; Glut, glucose transporter; GSK3ß, glycogen synthase kinase-3ß; HIF, hypoxia-induced factor; H2S, hydrogen sulfide; IGF, insulin-like growth factor; IL, interleukin; IRS, insulin receptor substance; ISCU, iron–sulfur clusters; NCOA4, nuclear receptor coactivator; NGF, nerve growth factor; NT, neurotensin; oxP, oxidative phosphorylation; rTMS, repetitive transcranial magnetic stimulation; TK, tyrosine kinase; TRK B, tropomysin-related kinase B; VEGF, vascular endothelial growth factor.

Under physiological conditions, cell metabolism depends on a continuous supply of glucose. Its uptake is strictly regulated and depends on glucose transporters (Gluts), which are stored in vesicles beneath the plasma membrane. Hormones, growth factors, cytokines, or neurotransmitters (Figure 3) induce the fusion of these vesicles with the membrane and the presentation of glucose transporters on the cell surface via AKT kinase activation.

The family of glucose transporters consists of 14 members [50]. Glut1, Glut3, and Glut4 play a predominant role in glucose supply and metabolic homeostasis. While Glut1 is a ubiquitous glucose transporter in most cells, Glut4 mediates insulin-dependent glucose uptake. Glut3 has the highest affinity for glucose of all glucose transporters and is predominantly expressed in neurons. In addition, much of the imported glucose is used by glia cells to glycolytically produce monocarboxylates like lactate, which fuel oxidative phosphorylation in the neuronal mitochondria [51].

The serine/threonine kinase Akt initiates a cascade of phosphorylations that lead to the translocation of Glut-containing vesicles and their fusion with the cell membrane. Three isoforms of Akt kinase regulate glucose uptake. Akt1 is the main isoform in all tissue cells, despite neuronal cells, which express the Akt3 isoform. Akt2 manages the insulin-induced glucose uptake through Glut4 and thereby guarantees basic glucose supply, even in the brain [4].

In addition to glucose import, Akt favors the storage of glucose as glycogen in astrocytes by inactivating glycogen synthase kinase-3 (GSK3), also known as tau protein kinase. This enzyme is associated with stress responses, neuronal apoptosis, and hyperphosphorylation of tau, which forms neurofibrillary tangles—a hallmark of AD. Impaired cerebral glucose metabolism triggers hyperphosphorylation of tau [52]. If Akt is active, GSK3 is inactive (Figure 3).

While the human brain is only 2% of body mass, it accounts for 20% of glucose utilization at rest [53]. Therefore, it is particularly vulnerable to glucose deprivation. Beside dementia, a variety of neurodegenerative conditions are associated with changes in glucose supply [54,55,56,57], and a low brain glucose metabolism rate is already initiated decades before the clinical onset of dementia [58,59].

Glucose, an essential energy substrate for the brain, travels in the blood and must traverse the blood–brain barrier via Glut1 to supply neuronal cells. In AD, decreased Glut1 and Glut3 levels in the brain limit glucose uptake [60], and positron emission tomography reveals that glucose utilization is dramatically lower in AD compared to a healthy brain [61,62]. Carriers of the epsilon4 allele of the *apolipoprotein E (ApoEε4)* gene, which is the major genetic risk factor for AD, display a regional pattern of cerebral glucose hypometabolism decades prior to disease onset [63].

In a transgenic mouse model of AD, which produces extracellular amyloid ß in the brain, reduced Glut1 levels on the surface of astrocytes weakened their ability to serve as a glucose conduit between capillaries and neurons [64,65].

Another transgenic mouse model carrying the human APOEε4 isoform displayed a reduction of about 30% in glucose transport through the blood–brain barrier compared with mice carrying the APOEε2 variant, although Glut1 expression in brain capillaries was unchanged [66]. In line with these results, Zhang observed high neuronal glycolytic activity in APOEε2 transgenic mice but low activity in APOEε4 mice, probably due to the limited glucose uptake [67]. ApoE also influences brain iron homeostasis. ApoE ko mice showed increased levels of amyloid ß, interleukin 6, and brain iron due to increased uptake and reduced ferroportin 1-mediated iron export [68]. Although the exact underlying mechanism remains unclear, disturbed insulin/Akt signaling seems to be involved [69].

Depression is also linked to abnormal energy metabolism. Kahl et al. [70] observed strong DNA methylation of the Glut1 promotor in leukocytes from depressive patients. DNA methylation impairs the gene expression of Glut1, leading to lower glucose uptake at the blood–brain barrier. Instead, insulin, glucose, cortisol, IL-6, and IL-1ß are elevated to counteract glucose deficiency [4]. While insulin and proinflammatory cytokines can activate further pathways for glucose uptake, cortisol restricts cellular energy consumption [71], interferes with Glut1 recycling via PTEN [72], and increases hepatic gluconeogenesis and blood glucose. Cytokine and hormone levels normalized after 6 weeks of treatment with antidepressants. In line with these findings, Stapel et al. [73] observed increased glucose uptake in blood cells under incubation with the antidepressant fluoxetine.

Insulin resistance, or type 2 diabetes mellitus, often triggered by obesity, is another factor that restricts glucose availability [74,75]. Therefore, it contributes to hypertension [76], osteoporosis [77], and AD [78]. Diabetic patients have a 50% increased risk of developing AD. Decreased sensitivity to insulin leads to hyperinsulinemia and hyperglycemia but reduced cellular glucose uptake. As shown in Figure 3 (magenta circle), insulin stimulates its own internal signaling cascade (insulin receptor-Akt2-Glut4) that results in the presentation of Glut4 on the cell surface. Disrupted insulin signaling disturbs the cellular energy metabolism by downregulating glucose transporters [79]. In line with these results, leukocytes from diabetic patients present low numbers of Glut3 and Glut4 on the plasma membrane [80]. The same was shown for the neuronal cells of obese mice on a high-fat diet [81]. In addition, insulin receptor ß-chain expression was reduced [82]. Thus, obesity lowers energy status in the brain despite chronic hypercaloric nutrition [34].

## 5. Cellular Mechanisms to Cope with the Energy Crisis

### 5.1. Integrated Stress Response

Reduced glucose uptake triggers intracellular ER stress and upregulation of the amyloidogenic ß-secretase BACE-1 [83,84] not only in AD but also in metabolic diseases, including type 2 diabetes, obesity, and cardiovascular disease [85]. One target protein of BACE-1 is the amyloid precursor protein (APP). Cleavage of APP by BACE-1 yields amyloid ß. This fragment helps to increase glucose uptake by activating the insulin-like growth factor receptor/Akt/Glut pathway under both basal and depolarizing conditions [86,87,88], but unfortunately, it tends to aggregate in the brain.

Plasma amyloid β levels are also elevated in patients with cardiometabolic diseases, obesity, type 2 diabetes, and heart failure [89,90]. In patients with depression, serum amyloid P, a constituent of amyloid deposits, is elevated [91]. In animal studies, injection of monomeric amyloid ß rescued glucose consumption in the brain of AD transgenic mice, while injection of oligomeric amyloid β caused cognitive impairment and reduced glucose availability due to impaired translocation of the insulin-regulated glucose transporter Glut4 [92].

Glucose deprivation initiates the integrated stress response, leading to hydrogen sulfide (H_2_S) production [4,93,94] (Figure 3). H_2_S is cytoprotective, prevents hyperphosphorylation of tau [95], and regulates energy production in mammalian cells under stress conditions [96,97] (Figure 3). Endogenous H_2_S in the brain is generated mainly by the cystathionine β-synthase, and its concentration is up to threefold higher in the brain than in serum [98]. In the brains of AD patients, H_2_S levels are low [99], while their plasma levels of homocysteine are increased. Cystathionine β-synthase diverts homocysteine to the biosynthesis of cysteine and the production of H_2_S. Mice or patients lacking this enzyme suffer from hyperhomocysteinemia and mental retardation [100]. In transgenic AD mice, treatment with the H_2_S donor GYY4137 improved cognition [95].

### 5.2. Inflammation

Energy crises in the brain are known to be induced by a reduction in glucose uptake, which may be ascribed to the diminished expression of cerebral glucose transporters due to impaired Akt signaling [101]. Reduced Glut1 presentation at the blood–brain barrier is counteracted by activated immune cells [102]. They secrete vascular endothelial growth factor that re-induces Akt signaling (Figure 3) and thereby restores cerebral glucose metabolism and preserves cognitive functions [103]. Thus, low chronic inflammation—linked to depression, hypertension, cardiovascular disease, osteoporosis, or obesity—can promote glucose uptake in undersupplied tissues. Diabetes even increases these inflammatory reactions.

Prolonged glucose or ATP depletion promotes a cascade of reactions leading to the formation of inflammasomes and the secretion of the proinflammatory cytokine interleukin 1ß [104]. It upregulates Glut1 [105], but also attracts activated immune cells. The glycolytic metabolism of these cells is linked to a high consumption of glucose [106]. Therefore, the inflammation might turn from a beneficial to a detrimental process, thereby increasing the energy crisis. Anti-inflammatory medications might slow down disease progression, but they do not stop energy depletion in the affected tissue. Moreover, in the brain, amyloid ß triggers metabolic reprogramming towards glycolysis during activation of microglia, and the increasing glucose deficiency leads to metabolically defective microglia and diminished immune responses in transgenic AD mice [107].

### 5.3. Disturbed Iron Metabolism

Mild hypoxic preconditioning has become a novel therapeutic target in the treatment of hypertension [108], myocardial infarction [109], cerebral ischemia [110], and depression [111]. It is even proposed as a potential application for patients with COVID-19 [112]. Hypoxia induces neuroprotection by reducing energy consumption [113] and prevents amyloid β accumulation in AD mice [114]. All these protective effects of hypoxia are mediated by the hypoxia-induced factor HIF1α.

Under physiological conditions, prolyl hydroxylase domain proteins (PHDs) sense molecular oxygen and mediate hydroxylation of the C-terminal transactivation domain of HIF1α, leading to ubiquitination and degradation of HIF1α. Under hypoxia, the hydroxylation of the alpha subunits is inhibited, thus stabilizing the HIF1α subunits [115]. HIF1α, together with HIF1ß, constitutes a transcription factor for several thousand genes and shifts cell metabolism towards oxygen-independent glycolysis [116]. Two of its target genes code for Glut1 and Glut3. Stronger expression of these genes allows for increased glucose uptake.

Surprisingly, glucose deprivation also activates HIF1α [117]. The mechanism of this activation relies on iron depletion because the HIF1α-destabilizing PHDs are Fe^2+^-dependent. Inhibition of Akt signaling, linked to reduced glucose uptake, simultaneously elevates intracellular ferritin levels [101]. In mitochondria, organelle-specific ferritin rescues mitochondrial iron overload and dysfunction [118]. During COVID-19 infection, hypomethylation of the ferritin heavy-chain gene underlies serum hyperferritinemia and low levels of serum iron in severely ill COVID-19 patients [119]. Prolonged Hif1α activity and higher miRNA210 expression downregulate the iron–sulfur cluster assembly machinery (ISCU) and divalent metal transporter 1 (DMT1) expression (Figure 3). ISCU is a scaffold protein that helps insert ferrous iron into the iron–sulfur clusters of proteins. Reduced ISCU levels are linked to decreased biogenesis of [Fe–S] clusters, leading to impaired protein maturation in the endoplasmic reticulum (ER) and increased ER stress [120]. Additionally, mitochondrial dysfunction is imminent because many proteins along the respiratory chain contain iron–sulfur clusters. The latest research shows that mitochondrial dysfunction is intimately associated with AD pathophysiology [121] and the development of type 2 diabetes [122].

The Iron importer DMT1 takes up nontransferrin-bound iron in many cells and also in mitochondria [123]. Its downregulation limits intracellular iron levels.

In contrast to hypoxia, glucose deprivation triggers additional interleukin 6 expression in immune cells [124], which subsequently induces hepatic hepcidin expression. Hepcidin, as a key regulator of iron metabolism, is pivotal in mediating the occurrence of anemia of chronic disease by blocking iron recycling by macrophages and duodenal iron absorption. Systemic hypoferremia is a part of the acute phase response of human infections and likely protects against extracellular infections. Excess iron is stored in the liver, and iron overload is diagnosed in 30% of patients with both non-alcoholic fatty liver disease and the metabolic syndrome [125].

Because iron is an essential cofactor for proteins involved in the tricarboxylic acid (TCA) cycle and electron transport in the mitochondria, for DNA synthesis, protein translation in the endoplasmic reticulum, activity of the cytochrome P450 system, and appropriate synthesis of the neurotransmitters serotonin, dopamine, and noradrenaline, iron sequestration in ferritin molecules must be reversible. The ferritin-specific autophagy adaptor nuclear receptor coactivator 4 (NCOA4), which is another HIF1α target, triggers ferritinophagy [126] to make intracellular, bound iron available again.

However, overactivation of NCOA4 and increased ferritinopathy liberate too much iron, ending in ferroptosis, an iron-provoked form of cell death characterized by intracellular lipid peroxide accumulation and oxidative membrane damage [127]. Iron deposits were previously shown in pancreatic ß cells from diabetic mice. The cells die by ferroptosis [128], as do liver cells [129] and kidney tubular cells [130] under diabetic conditions.

Ferroptosis also contributes to neurodegeneration in AD [131], although the exact mechanism is still a matter of debate. The iron-responsive element in the 5′-untranslated region of the APP mRNA points to a function of APP in iron homeostasis, i.e., the stabilization of the iron exporter ferroportin in the plasma membrane [132]. Shifting the balance from APP to Aß reduces iron export but increases the excess of unstored intracellular iron and the formation of reactive oxidative species.

**Figure 4:**
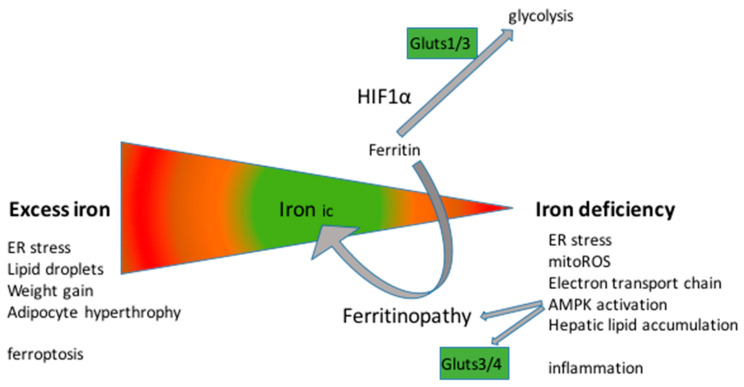
Cellular iron homeostasis. Under stress, ferritin reduces the free iron pool, which stabilizes HIF1α and impairs the activity of key electron transport chain and Krebs cycle enzymes. In turn, reduced ATP production leads to reactive oxygen species (ROS) production in the mitochondria. Excess ferritin production, leading to hyperferritinemia, causes inflammasome formation and the attraction of immune cells. In contrast, excess iron due to exaggerated ferritinopathy increases oxidative stress in the cytoplasm, lipid peroxidation, the formation of lipid droplets, and ferroptosis.

Intracellular iron metabolism is a double-edged sword. Insufficient levels of free iron disturb oxidative phosphorylation, while excessive amounts of iron cause oxidative stress and ferroptosis (Figure 4). A key physiological player in this regulation is H_2_S [133]. Alternatively, iron chelation [134,135] can help stabilize iron levels in the physiological range and thereby remit memory deficits and slow down disease progression. Indeed, some clinical trials show that the dosage of iron chelators should be finely tuned to avoid worsening the disease [136].

## 6. Protective Treatments

Iron depletion is an active process. The hepcidin-ferroportin pathway downregulates cellular iron import and limits gastrointestinal iron uptake. The efficacy of oral iron supplementation is doubtful because iron deficiency/anemia is a consequence of insufficient glucose uptake. Thus, the focus of treatment should be on the import of glucose or alternative substrates. Table 1 gives an overview of potentially helpful nutrients and drugs and their effects on glucose uptake and iron homeostasis.

### 6.1. Ketogenic Diet

The ketogenic diet is known as a non-pharmacological treatment for metabolic disorders as well as brain disorders. Fat serves as the main alternative energy source under a ketogenic diet (diet ratio of 4:1 fat to non-fat energy sources) and causes a metabolic shift from glucose to fat utilization, favoring fat oxidation, ketone production, and ketone use. The mitochondria easily adapt to the changed parameters. They preserve their functionality by increasing proteins in the TCA cycle and respiratory chain. Moreover, the biogenesis of mitochondria is observed on a ketogenic diet [137].

This diet maintains insulin signaling and prevents iron dyshomeostasis by downregulating iron import and elevating ferritin levels [138]. In patients with type 2 diabetes, the ketogenic diet has beneficial effects on weight loss and less glycosylated hemoglobin [139]. It also improves depression and psychotic symptoms [140], as well as cognitive symptoms associated with different neurodegenerative disorders [141], including AD [138]. In the brain, mainly astrocytes and neurons consume ketone bodies. Furthermore, a ketogenic diet increases Glut3 expression and glucose uptake in neurons [52].

### 6.2. HIF-Prolyl Hydroxylase Inhibitors (HIF-PHIs)

HIF-PHIs influence iron homeostasis through effects on transferrin, transferrin receptor expression, hepcidin, and other iron-related proteins [142]. They activate HIF1α without iron deprivation [143] in patients with heart failure and renal anemia. HIF-PHIs increase plasma hemoglobin levels by restoring physiological erythropoietin production in the kidney, while hepcidin levels tend to decrease [144]. Moreover, HIF stabilizers show adverse effects on the development of renal fibrosis, angiogenesis, and vascular calcification [145].

HIF-PHIs are already used in clinical practice, and ongoing research will define a role for these drugs beyond the management of anemia. The application of HIF-PHIs might strengthen the antiviral properties of the HIF signaling pathway in SARS-CoV-2 replication and other pathogens, thereby offering new therapeutic opportunities [146].

In HIF-prolyl-4-hydroylase2-ko mice, constitutive HIF1 activation preserves heart functions after myocardial infarction [115,147] and triggers osteoblast differentiation by the metabolic shift towards glycolysis [148]. HIF-PHIs further protect against obesity-related diseases and atherosclerosis [149,150,151,152,153].

In diabetic patients, hyperglycemia represses HIF1α induction under hypoxia and increases ROS production, which is suspected to trigger further diabetic complications [154]. In diabetic animal models, HIF stabilization by HIF-PHIs prevents a metabolic shift toward fat oxidation and rhabdomyolysis that preserves renal functions and accelerates wound healing [155,156]. Future studies will certainly uncover many more potential benefits of HIF-PHI treatment.

### 6.3. Metformin

Metformin is considered a first-line anti-diabetic medication for type 2 diabetes mellitus and one of the most commonly prescribed drugs in the world. Due to its positive effects on insulin resistance and glucose metabolism, which play an important role in AD, it was investigated as a treatment option for dementia. In animal models, metformin increased glucose uptake and attenuated amyloid ß production [157,158,159]. Although the exact mechanism underlying metformin treatment is not fully understood, it improves cellular energy homeostasis via AMPK/insulin receptor/Akt signaling (Figure 3). AMPK is an intracellular energy sensor. Its activity is inversely correlated with the cellular availability of glucose. Low intracellular ATP levels activate this pathway, which boosts neuronal autophagy and increases Glut4-mediated glucose uptake [160]. In addition, it has pleiotropic beneficial effects on depression [161,162] and inflammation [163]. Furthermore, it prevents diabetic as well as glucocorticoid-induced osteoporosis via activating AMPK [164]. A large meta-analysis recently revealed a significantly lower risk of major cardiovascular events under metformin treatment [165], and Molaei et al. regard this pathway as a novel therapeutic target for cardiovascular diseases [166]. Notably, iron chelation activates AMPK signaling [167]. Accordingly, metformin was demonstrated to primarily induce an iron starvation response [168] with increased expression of the *ferritin heavy-chain* gene. Consistent with these results, metformin improved anemia in a mouse model of polycystic kidney disease [169].

### 6.4. Lithium

Lithium is a common medication used to treat mania and bipolar disorder. Metabolic dysfunction, characterized by disturbed glucose metabolism and insulin resistance, is becoming increasingly recognized as important in the pathophysiology of bipolar disorder [170].

Harmstra et al. [171] provided evidence that a low dose of lithium also slows down the aging process and protects from age-related diseases, such as osteoporosis, AD, type 2 diabetes mellitus, cardiovascular disease, and chronic inflammation. Even clinical studies reveal a delay in neurodegeneration under lithium [172]. GSK-3ß is a well-known target of lithium, and chronic GSK3β overactivation has been shown to disrupt brain energy regulation via impairment of glucose metabolism and mitochondrial function. Recently, Ates et al. [173] presented evidence that neuroprotection by lithium depends on GSK3ß phosphorylation along the Akt pathway. Under drug treatment, hippocampal glucose uptake is enhanced by a strong increase in Glut3 [174]. Although Akt signaling is the main pathway to increase glucose uptake, involvement of AMPK/insulin receptor signaling is very likely—at least in the transgenic AD mouse.

### 6.5. Phytochemicals: Resveratrol, Gingko Biloba, Berberine, Curcumin, and Icariin

Nutritional interventions are often used to arrest or reverse age-related disorders. In particular, Ayurveda, an Indian traditional medical system, uses ingredients of natural origin, including whole plants or certain portions of plants and minerals, for therapeutic purposes. Different extraction methods have been shown to yield different compounds with specific biological effects, and combinations of phytochemicals may lead to potential novel potent therapies for age- and lifestyle-related disorders due to additive or synergistic effects. Fruits, berries, and herbs have plenty of ingredients, such as polyphenols, terpenes, flavonoids, catechines, glycosides, cerebrosides, lignans, and phytosterols, that trigger anti-inflammatory, antioxidant, anti-diabetic, and antiviral pathways [175]. Moderate consumption of red wine correlates with a lower risk for AD [176,177,178], osteoporosis [179], and cardiovascular disease [180].

Many phytochemicals possess high antioxidant activity. In addition, they target the nuclear factor E2-related factor2/heme oxygenase 1 (NRF2/HO-1) pathway to reduce oxidative stress and inflammation [181,182]. Either high intracellular radical levels—generated either by free heme molecules or dysfunctional mitochondria—or AMPK activation [183] induce the transcription factor NRF2 and its target genes *ferritin* and *hmox-1*, coding for heme oxygenase 1. This enzyme catalyzes the oxygenation of heme, thereby producing biliverdin, carbon monoxide, and reactive Fe^2+^, which is directly chelated by ferritin. The other cleavage products scavenge radicals as well [184]. Besides its important role in maintaining cellular redox balance, Nrf2 is also engaged in the regulation of iron metabolism, contributing to maintaining iron bioavailability and ferroptosis resistance [185].

### 6.6. Resveratrol

Resveratrol, the ingredient in red wine, is a stilbenoid polyphenol with multiple bioactivities. Its supplemental intake mimics caloric restriction. It binds to Glut1 and reduces glucose uptake [186]. This transient intracellular glucose deprivation confers a preconditioning effect by triggering different survival pathways. While low levels of resveratrol induce sirtuin 1, which, in turn, promotes membrane localization and activation of the AKT2 isoform, higher levels of resveratrol promote the same pathway as metformin [187]. The final aim in either the sirtuin 1/Akt2 pathway or the AMPK/Glut4 pathway is increased glucose uptake. Besides resveratrol, metformin, empagliflozin, and bexarotene show cardioprotective effects linked to the AMPK pathway [188]. In addition, resveratrol prevents hepatic iron overload and nonalcoholic fatty liver disease in rats by reducing iron uptake and increasing iron export [189,190]. However, Sangouni et al. [190] found no improvement in cardiovascular indices or hepatic steatosis in diabetic patients treated with resveratrol for 8 weeks.

### 6.7. Gingko Biloba

The standard extract of *Gingko biloba* leaves contains a mix of beneficial catechins and procyanidins, together with flavonoid glycosides and terpene trilactones. It has anti-osteoporotic [191] as well as anti-inflammatory properties [192]. In the brain, it reduces amyloid ß toxicity and destabilizes neurofibrils [193]. Moreover, it reduced zinc and hyperhomocysteinemia-linked tau hyperphosphorylation [194,195]. These beneficial effects might all result from improved insulin signaling [196] and iron scavenging. *Ginkgo biloba* extracts strongly chelate ferrous ions [197] and increase NRF2 expression, preventing ferroptosis and nonalcoholic fatty liver disease in obese mice [198].

### 6.8. Berberine

In East Asia, the isoquinoline alkaloid berberine is used to cure a wide range of diseases, including cognitive decline, obesity, type 2 diabetes mellitus, cardiovascular disease, atherosclerosis, and osteoporosis [199]. Although no clinical studies have been carried out, berberine shows significant improvement in memory in several animal models of AD [200]. Besides its anti-inflammatory and anti-oxidative effects, berberine strongly improves glucose uptake in the brain [201] by increasing Glut3 expression and Glut1 activity [202].

### 6.9. Curcumin

Curcumin is a polyphenolic diketone from turmeric with antioxidant, anti-inflammatory, anti-cancer, anti-microbial, and hypoglycemic properties [203]. It reduces blood glucose and oxidative stress and increases plasma insulin in diabetic mice [204]. Consistent with these results, the insulin/Akt2/Glut4 pathway was upregulated in skeletal muscle cells [205] (Figure 3, magenta circle). Moreover, curcumin is a brain-permeable iron chelator [206], has a high binding affinity for amyloid β, and directly mitigates amyloid ß aggregation [207,208]. However, its anti-cancer properties might rely on the inhibition of the transport activity of Glut1 [209].

### 6.10. Icariin

The flavonoid compound icariin is a bioactive constituent of *Herba Epimedii*. There is striking evidence that icariin ameliorates the comorbidities of type 2 diabetes mellitus: cardiomyopathy [210], retinopathy [211], wound healing [212], osteoporosis [213], chronic kidney disease [214], and obesity [215]. It even improves pancreatic functions and hyperglycemia [216]. In liver cells that are unresponsive to insulin, increased activity of the protein tyrosine phosphatase 1B dephosphorylates the insulin receptor [217]. Inhibition of this enzyme by icariin increases the amount of Glut4, phosphorylates and thereby activates insulin receptors and glycogen synthase.

In the brains of transgenic AD mice, icariin restored insulin signaling and the phosphorylation levels of the insulin receptor ß chain, insulin receptor substrate, GSK3ß, and Akt [218]. Glut1 and Glut3 levels were also markedly increased. Icariin improved learning and memory impairments in animal models of AD and depression, although it cannot effectively penetrate the blood–brain barrier [219]. The mechanism of these indirect effects is still unknown, but changes in the systemic iron metabolism might be involved because icariin increases hepatic hepcidin expression [220]. Reduced iron availability might also be linked to suppression of ferroptosis in synoviocytes [221] and activation of HIF1α in chondrocytes [222]. In addition, icariin protects against iron-overload-induced bone loss [223].

## 7. Conclusions

Aging is linked to a slow decrease in estrogen and growth factors, low-grade inflammation, and anemia [4]. Anemia is even recognized as a risk factor for increased morbidity and mortality [224]. Low-grade systemic inflammation with higher levels of interleukin 6 activates hepcidin, thus impairing the duodenal absorption of iron and the release of iron from macrophages and hepatocytes, leading to a decline in circulating iron and increased iron storage in the liver. High serum CRP and ferritin levels point to a fat liver with iron deposits and anemia [225]. Tomczyk et al. [226] proved the direct link between glucose availability, interleukin 6, and iron metabolism. In their study, glucose but not fructose supplementation abrogated exercise-induced increases in hepcidin and IL6 levels. On the contrary, an unhealthy diet with a lot of sweets, a sedentary lifestyle, and obesity are linked to decreased cellular glucose import and elevated levels of proinflammatory cytokines and serum hepcidin.

Anti-inflammatory therapies or oral iron supplementation decelerate disease progression, but they do not treat the root cause of energy depletion. Instead, improved Akt signaling and glucose uptake must be the final aim. In the cell, this is achieved in two different directions: HIF1α increases the expression of glucose transporters, while the energy sensor AMPK increases glucose import via the Akt2/Glut4 pathway. Both proteins are activated by iron depletion [116,167], thereby causing iron deficiency and, further on, anemia. Supporting these physiological approaches to activate HIF1α by HIF-prolyl hydroxylase inhibitors or AMPK by metformin [164] might prevent anemia [169] and protect from mitochondrial dysfunction.

In a retrospective study, Silverburg et al. demonstrated that subcutaneous erythropoietin and intravenous iron not only improved anemia but also cardiac and renal functions in patients with heart failure [227]. These patients often have an iron-deficient state, which can limit erythropoiesis in erythroid precursors and ATP production in cardiomyocytes.

Neurodegenerative diseases most often present with cellular iron deficiency, energy depletion, and inflammation. However, the underlying cellular glucose deprivation remains obscure. The brain is most susceptible to glucose deprivation. Only astrocytes have glycogen stores. Glucose transport into the brain is near its maximum and relies on saturable transporters at the blood–brain barrier. Almost all of the imported glucose is used. Decreased glucose transport across the blood–brain barrier and into neurons—caused by obesity, older age, hyperglycemia, insulin resistance, or reduced neurotransmitter signaling—can drive a starving brain into dysfunction. Tkacheva et al. could even distinguish patients with dementia from patients with minor cognitive impairment due to the severity of anemia, depression, and malnutrition, among other parameters [228].

The occurrence of iron deficiency/anemia in the development of all the diseases presented in the graphical abstract is at best noticed, although it might further help to clarify more fundamental mechanisms involved in disease progression. Recognizing a significant drop in hemoglobin as a sign of local glucose deprivation should start a clinical search for the underlying causes in order to prevent a vicious cycle where different diseases, such as insulin resistance and iron deficiency, affect each other (graphical abstract; [229]). Nutrition or the precautionary intake of phytochemicals with their anti-inflammatory effect might also help to improve cellular glucose import (Table 1). Iron deficiency and inflammation then resolve on their own.

## Figures and Tables

**Table 1:** Overview of possible protective treatments that improve cellular glucose import and iron deficiency. It must be noted that phytochemicals are extensively studied in regard to their anti-inflammatory and anti-oxidative effects, but less is known concerning the iron metabolism. Arrows indicate up- or downregulation of glucose transporters and proteins linked to iron metabolism. The question marks indicate that the effects are not known.

	Treatment	Pathway	Substrate	Energy Production	Glucose Transporter	Effects on Iron Metabolism
**pharmaceuticals**	metformin	AMPK activation	glucose	respiratory chain	Glut4↑	Ferritin ↓
	lithium	GSK3ß inhibition	glucose	respiratory chain	Glut1/3↑	?
	HIF-PHIs	HIF1α activation	glucose	glycolysis	Glut1/3↑	Hemoglobin↑
**nutrition**						
diet	Ketogenic diet	fat oxidation	fat, glucose	respiratory chain	neurons: Glut3↑	serum ferritin, hepcidin ↓
phytochemicals	resveratrol low dose	Sirtuin 1 activation	glucose	respiratory chain	Glut1/3↑	Ferroportin ↑
	resveratrol high dose	AMPK activation	glucose	respiratory chain	Glut4↑	
	Gingko biloba	NRF2/HO-1 activation	glucose	respiratory chain	?	iron chelation
	berberine	NRF2/HO-1 activation	glucose	respiratory chain	Glut1 ↓	?
	curcumin	NRF2/HO-1 activation	glucose	respiratory chain	Glut1/3↑	iron chelation
	icariin	HIF1α activation	glucose	glycolysis	Glut1/3/4↑	hepcidin ↑
